# Distribution and pyrethroid resistance status of *Aedes aegypti* and *Aedes albopictus* populations and possible phylogenetic reasons for the recent invasion of *Aedes aegypti* in Nepal

**DOI:** 10.1186/s13071-020-04090-6

**Published:** 2020-04-22

**Authors:** Hitoshi Kawada, Kyoko Futami, Yukiko Higa, Ganesh Rai, Takashi Suzuki, Shiba Kumar Rai

**Affiliations:** 1 Institute of Tropical Medicine, Nagasaki University, Nagasaki, Japan; 2 Departmanet of Medical Entomology, National Institute of Infectious Diseases, Tokyo, Japan; 3 Shi-Gan International College of Science and Technology, Kathmandu, Nepal; 4 Faculty of Health Science, Kobe-Tokiwa University, Kobe, Japan; 5 Division of Medical Informatics and Bioinformatics, Kobe University Hospital, Kobe, Japan; 6 Research Division, Nepal Medical College, Gokarneswor, Kathmandu, Nepal

**Keywords:** Nepal, *kdr*, Pyrethroid, Resistance, *cox*1, Invasion

## Abstract

**Background:**

When the first systematic list of mosquitoes in Nepal was reported in 1990, there was no description of *Aedes aegypti* (L.), while *Aedes albopictus* (Skuse) has been included in the *Stegomyia* subgroup since the 1950s. The first record of *Ae. aegypti* in Nepal was reported in 2009, suggesting some coincidence between the invasion of this species and the first record of dengue fever in Nepal in 2006.

**Results:**

We performed a field survey of the distribution and insecticide susceptibility of *Ae. aegypti* and *Ae. albopictus* in Nepal in 2017 and 2018. Mosquito larvae were collected from used tires located along the streets of Kathmandu, Bharatpur and Pokhara, and a simplified bioassay was used to assess the susceptibility of the larvae to pyrethroid insecticides using *d*-allethrin. The presence or absence of point mutations in the voltage-gated sodium channel was also detected by direct sequencing. V1016G was detected at a high frequency and a strong correlation was observed between the frequencies of V1016G and susceptibility indices in *Ae. aegypti* populations. F1534C was also detected at a relatively low frequency. In *Ae. albopictus* populations, susceptibilities to *d*-allethrin were high and no point mutations were detected. Analysis of the cytochrome *c* oxidase subunit 1 (*cox*1) gene was performed for assessing genetic diversity and the existence of two strains were identified in *Ae. aegypti* populations. One consisted of 9 globally-distributed haplotypes while the other was derived from an African haplotype.

**Conclusions:**

The high pyrethroid resistance, high V1016G frequency, and relatively low quantity of insecticides used to control dengue vectors in Nepal may have resulted in only weak selection pressure favoring insecticide resistance and could support the hypothesis that this species has recently been introduced from neighboring Asian countries where pyrethroid resistance is relatively widespread.
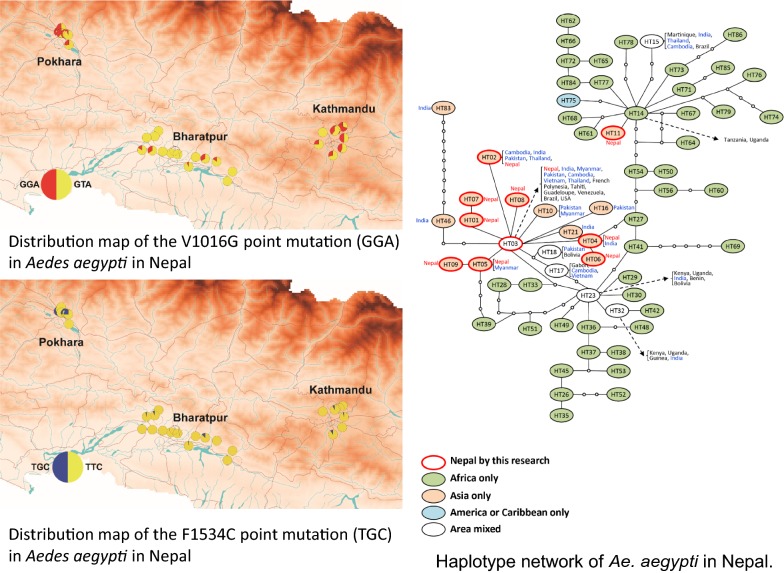

## Background

The first systematic survey of mosquitoes in Nepal was performed by Darsie & Pradhan [1] in 1990. Interestingly, there was no record of *Aedes aegypti* (L.), while *Aedes albopictus* (Skuse) had been observed in Nepal and recorded as part of the subgenus *Stegomyia* since the 1950s [1, 2]. The first record of *Ae. aegypti* in Nepal was reported only recently in 2009 [3], and the first case of dengue virus infection was reported in 2004 in a Japanese volunteer working in Chiwan district [4]. Therefore, a cause-and-effect relationship between the invasion of *Ae. aegypti* by 2009 and the first outbreak of dengue fever (DF) in 2006 [5] appears to be well correlated. In Nepal, *Ae. aegypti* and *Ae. albopictus* are reported to be common in the Kathmandu Valley (1350 m above sea level) but are rarely found in Dhunche in the Rasuwa district (1750 to 2100 m above sea level) [6]. It was thus concluded that the expansion of these mosquitoes even to higher altitude might be attributable to environmental and climate change which has been occurring for several decades in Nepal [7]. Heightened transportation and the urbanization of new areas are also thought to be the major causes of the expansion of mosquito populations [8].

Tragically, dengue fever has been rife in Nepal and 14,662 people (from 68 of 77 districts in Nepal) have been diagnosed with dengue fever with total of six deaths since May 13th 2019 [9]. The highest number of dengue cases (7151) was reported in Bagmati Province including the capital city (Kathmandu) in which 1583 cases have been confirmed. This could influence the national campaign of “Visit Nepal Year 2020” which targeted two million foreign tourists [9]. The Himalayan Times (August 29th 2019) reported that “The present dengue outbreak is the longest in duration, largest in terms of cases and most devastation in Nepali history, one that is unfortunately still spreading aggressively in Kathmandu and across the country” (https://thehimalayantimes.com/opinion/dengue-in-kathmandu-threat-of-larger-outbreak/). Fogging of insecticides (deltamethrin) has been carried out in urban area for controlling vector mosquitoes (*Ae. aegypti* and *Ae. albopictus*) (GR, unpublished data).

In light of this, we performed field surveys of the distribution and insecticide susceptibility of *Ae. aegypti* and *Ae. albopictus* populations in 2017 and 2018. The aim of this study was to provide entomological information on the risk status of DF and chikungunya in Nepal based on vector dynamics, as well as to potentially aid in the control of mosquitoes. The distributions of *Ae. aegypti* and *Ae. albopictus* were mapped and the extent of insecticide resistance in each of the populations was assessed using simplified knockdown assays in larvae as well as the detection of point mutations in the voltage-gated sodium channel. The possible pathways of pyrethroid-resistant *Ae. aegypti* migration from neighboring countries are also discussed.

## Methods

### Collection of *Aedes* larvae from used tires and the application of a simplified knockdown bioassay

As the main land of Nepal is located in the Himalayas, but also includes parts of the Indo-Gangetic Plain, we thought that the elevation of mosquito sampling sites might affect climate, species composition and insecticide susceptibility; therefore, we selected three cities at different elevations (Kathmandu, Pokhara and Bharatpur). By driving through the main roads in the three cities, we collected mosquito larvae from used tires during 20–24 August 2017 (Kathmandu and Bharatpur), and 12–13 September 2018 (Pokhara). Collection sites in Kathmandu were located 1300 m above sea level and experienced a mild temperate climate (average monthly temperature varied between 3–29 °C). Collection sites in Pokhara were located 800–900 m above sea level and experienced a warmer climate than sites in Kathmandu (average monthly temperature varied between 8–32 °C). Collection sites in Bharatpur were located 200 m above sea level and experienced a hot season during March and July (average monthly temperature varied between 11–36 °C). Kathmandu, Pokhara and Bharatpur have a 4-month rainy season from June to September with a maximum monthly precipitation of 185 mm, 213 mm and 225 mm, respectively. The geographical locations of collection sites were recorded using a global positioning system (GPS). Mosquito larvae were collected from tires using a net.

Simplified bioassays were used to assess the knockdown of *Aedes* larvae and were performed on the day of collection, according to the methods of Kawada et al. [10] and using two concentrations of *d*-T_80_-allethrin (0.1 and 0.4 ppm). Knockdown of mosquito larvae were judged when larvae sank to the bottom of the glass vial and could not swim, or were paralyzed. KT_50_ (the time required for 50% knockdown of larvae) was recorded for each concentration regime and knockdown was scored according to the following six categories: score 1, < 5 min; score 2, 5 to < 10 min; score 3, 10 to < 15 min; score 4, 15 to < 20 min; score 5, 20 to < 30 min; and score 6, ≥ 30 min. Susceptibility indices were calculated by multiplying scores at 0.1 ppm and 0.4 ppm. Therefore, mosquito larval group with a susceptibility index of 1 was judged as the most susceptible to *d*-allethrin, while those with an index of 36 was considered to be the least susceptible to *d*-allethrin [10]. After bioassays, we identified each larva according to the keys of Rattanarithikul et al. [11]. The larvae were then individually stored in 100% ethanol in a 1.5 ml Eppendorf tube prior to subsequent analysis.

### Analysis of the frequency of point mutations

PCR and direct DNA sequencing were used to detect the presence of point mutations at 982L, 989S, 1011I, 1014L, 1016V and 1534F in *Ae. aegypti* and *Ae. albopictus* according to the methods previously described by Kawada et al. [10]. Sample size of mosquitoes at each city for genetic analysis was aimed at more than 60 according to Bashalkhanov et al. [12]. *Aedes aegypti* DNA was initially amplified using the primers AaSCF1 (5’-AGA CAA TGT GGA TCG CTT CC-3’) and AaSCR4 (5’-GGA CGC AAT CTG GCT TGT TA-3’) for S989P, I1011M, L1014F and V1016G, or AaSCF7 (5’-GAG AAC TCG CCG ATG AAC TT-3’) and AaSCR7 (5’-GAC GAC GAA ATC GAA CAG GT-3’) for F1534C. *Aedes albopictus* DNA was initially amplified using the primers AaSCF20 (5’-GAC AAT GTG GAT CGC TTC CC-3’) and AaSCR21 (5’-GCA ATC TGG CTT GTT AAC TT G-3’) for S989P, I1011M, L1014F and V1016G, or AaSCF7 (5’-GAG AAC TCG CCG ATG AAC TT-3’) and AaSCR7 (5’-GAC GAC GAA ATC GAA CAG GT-3’) for F1534C. DNA sequencing of *Ae. aegypti* was carried out using the primers AaSCF3 (5’-GTG GAA CTT CAC CGA CTT CA-3’) and AaSCR6 (5’-CGA CTT GAT CCA GTT GGA GA-3’) for S989P, I1011M, L1014F and V1016G, or AaSCR8 (5’-TAG CTT TCA GCG GCT TCT TC) for F1534C. DNA sequencing of *Ae. albopictus* was carried out using the primers AaSCF3 (5’-GTG GAA CTT CAC CGA CTT CA-3’) and AaSCR22 (5’-TTC ACG AAC TTG AGC GCG TTG-3’) for S989P, I1011M, L1014F and V1016G, or AaSCR8 (5’-TAG CTT TCA GCG GCT TCT TC-3’) for F1534C. The targeted amino acid replacement was analyzed using the software MEGA 6.0 (http://www.megasoftware.net/) and ATGC for Windows version. 9.0.0 (Genetyx Corporation Tokyo, Japan). The unique DNA haplotype sequences were deposited in the GenBank database under the accession numbers LC485541-LC485548.

### Phylogenetic analysis

Analysis of the cytochrome *c* oxidase subunit 1 (*cox*1) gene was performed for assessing the genetic diversity of *Ae. aegypti*. DNA extracts of the mosquito samples were used as templates to amplify fragment of the *cox*1 gene. Initial PCR amplification was carried out using the primers LCO1490 (5’-GGT CAA CAA ATC ATA AAG ATA TTG G-3’) and HCO2198 (5’-TAA ACT TCA GGG TGA CCA AAA AAT-3’) [13]. PCR was performed using TaKaRa Ex Taq (Takara Bio Inc., Kusatsu, Japan). The PCR mixture contained 1.0 µl of 10× EX Taq buffer, 0.8 µl of 2.5 µM dNTP mixture, 0.05 µl of 5 U/µl Ex Taq HS, 0.4 µl of each 2.5 µM primer, and 1.0 µl of DNA template in a total volume of 10 µl. PCR cycling conditions were as follows: initial denaturation at 94 °C for 1 min; 35 cycles at 94 °C for 10 s, 45 °C for 30 s, and 72 °C for 60 s; and a final extension step at 72 °C for 10 min. The amplified fragments were purified with ExoSAP-IT (USB Corporation, Cleveland, USA) at 37 °C for 30 min, and then at 80 °C for 15 min. DNA sequencing was carried out using the primers LCO1490 and HCO2198 in the same manner as previously described.

Available *cox*1 gene sequences of mosquito species were downloaded from GenBank and aligned with MEGA 7 [14] and ATGC for Windows version. 9.0.0 (Genetyx Corporation). The unique DNA haplotype sequences were deposited in the GenBank database under the accession numbers LC485549-LC485558, LC489421-LC489422. Sequences with double peaks were identified as suspected nuclear mitochondrial pseudogenes (NUMTs) and removed from subsequent analyses. A sequence with an insertion was removed from the alignment because the insertion drastically changed amino acid sequences, suggesting it may be a NUMT. To avoid including incorrect indels, sequences were trimmed from both sides and edited to 525–593 bp. Haplotypes were determined based on the pairwise differentiation of nucleotides calculated with MEGA 7.

### Data analysis

A Chi-square test was conducted to analyze the *Aedes* species distributions in each city, and allelic point mutation frequencies (V1016G and F1534C) using JMP Pro 14.0.0 (SAS Institute Japan Inc. Tokyo, Japan). One-way analysis of variance was performed for evaluation of the susceptibility indices using JMP Pro 14.0.0. Univariate analysis was used to assess the correlation between susceptibility indices and the frequencies of point mutations (arcsine-transformed) using JMP Pro 14.0.0.

Haplotype diversity (Hd) [15] and nucleotide diversity (π) [16] were calculated using Excel and DnaSP [17]. Tajima’s *D* was calculated using DnaSP based on 576 bp sequences with missing sites excluded. To construct the haplotype network, *cox*1 sequences from around the world were obtained from previous studies [18–30] and GenBank (Additional file 1: Table S1). The haplotype network was constructed using TCS v.1.21 [30] based on data from Nepal, Myanmar and other global haplotypes measuring 589–593 bp. Parsimony threshold probability was set at 95%.

## Results

### Pyrethroid susceptibility and point mutations in the voltage-gated sodium channel in *Ae. aegypti* and *Ae. albopictus*

**Fig. 1:**
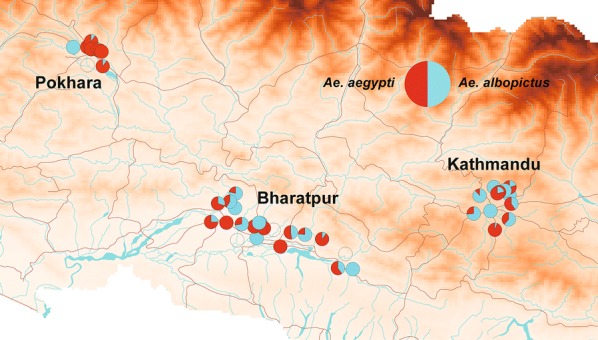
Distribution map of *Aedes aegypti* and *Aedes albopictus* larvae in Kathmandu, Bharatpur and Pokhara. The larvae were collected from used tires located along the roadside. Circles indicate the species composition of the two species at the collection site. The data were plotted on a shape file map (DIVA-GIS, http://www.diva-gis.org/gdata) using QGIS 3.4 (https://www.qgis.org/ja/site/forusers/download.html)

Figure 1 shows the species distribution patterns of both *Ae. aegypti* and *Ae. albopictus* larvae collected from used tires in three cities in Nepal. Eight hundred and eighty larvae were collected. Among them 442 were *Ae. aegypti*, 359 were *Ae. albopictus*, and 79 were other species including *Culex* sp., *Armigeres* sp. and other unidentifiable species. *Aedes aegypti* and *Ae. albopictus* larvae were identified in 33 and 35 of the 45 collection sites, respectively, indicating the predominance of these mosquitoes in the collection sites. *Aedes aegypti* was dominant in Pokhara relative to the other cities (Bharatpur and Kathmandu) (*χ*^2^ = 75.4, *df* = 2, *P* < 0.0001).

**Fig. 2:**
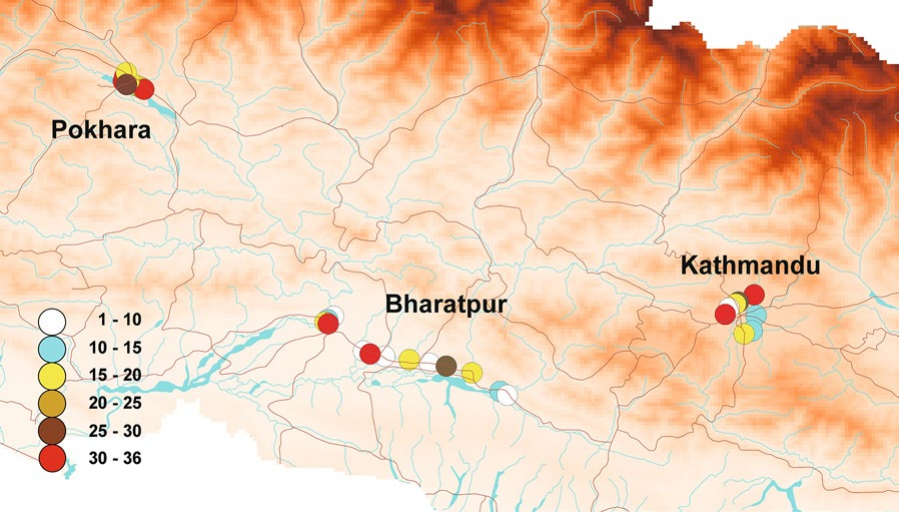
Distribution map of susceptibility indices in *Aedes aegypti* larvae. Susceptibility indices were determined using a simplified knockdown bioassay. The larger the index, the lower the susceptibility to *d*-allethrin. The data were plotted on a shape file map (DIVA-GIS, http://www.diva-gis.org/gdata) using QGIS 3.4 (https://www.qgis.org/ja/site/forusers/download.html)

**Fig. 3:**
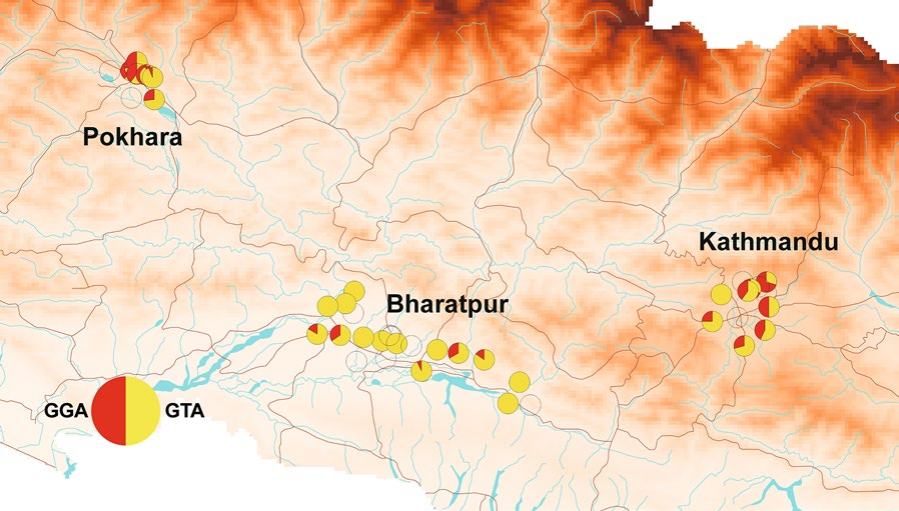
Distribution map of the V1016G point mutation in *Aedes aegypti* larvae. Circles indicate the allelic composition of the point mutations (GGA)/wild types (GTA) at collection points. The data were plotted on a shape file map (DIVA-GIS, http://www.diva-gis.org/gdata) using QGIS 3.4 (https://www.qgis.org/ja/site/forusers/download.html)

**Fig. 4:**
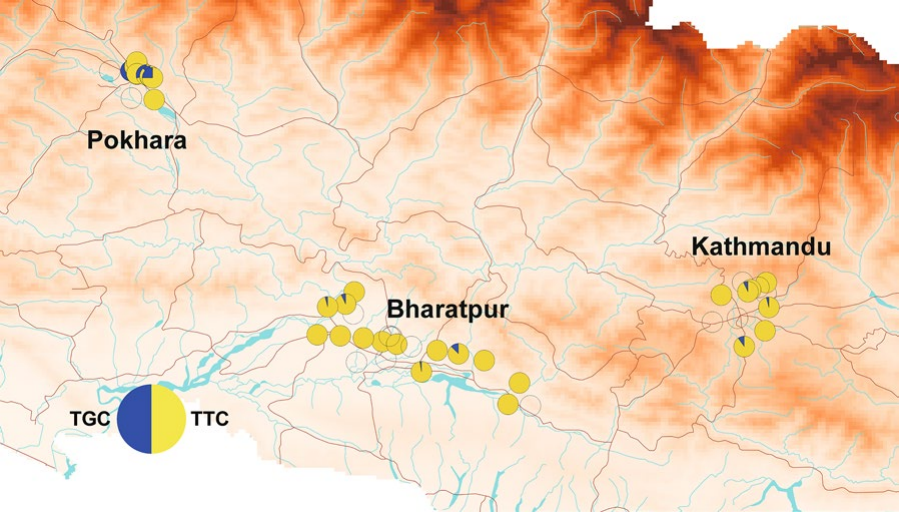
Distribution map of the F1534C point mutation in *Aedes aegypti* larvae. Circles indicate the allelic composition of the point mutations (TGC)/wild types (TTC) at collection points. The data were plotted on a shape file map (DIVA-GIS, http://www.diva-gis.org/gdata) using QGIS 3.4 (https://www.qgis.org/ja/site/forusers/download.html)

**Fig. 5:**
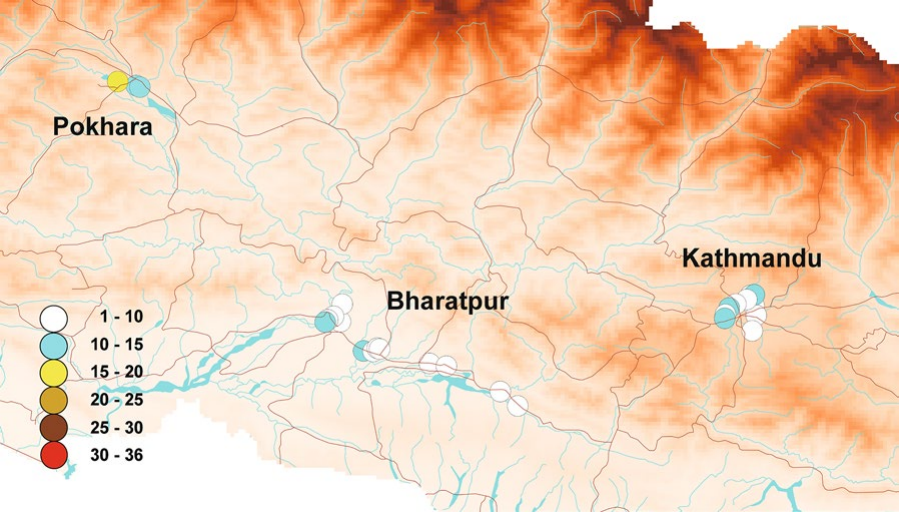
Distribution map of susceptibility indices in *Aedes albopictus* larvae. Susceptibility indices were determined using a simplified knockdown bioassay. The larger the index, the lower the susceptibility to *d*-allethrin. The data were plotted on a shape file map (DIVA-GIS, http://www.diva-gis.org/gdatan) using QGIS 3.4 (https://www.qgis.org/ja/site/forusers/download.html)

Figure 2 shows the distribution of the susceptibility indices of *Ae. aegypti* larvae, as determined by simplified knockdown bioassays. High susceptibility indices (> 30) were recorded in 3 of the 13 collection sites in Bharatpur, 3 of the 8 collection sites in Kathmandu, and 5 of the 11 collection sites in Pokhara (Tables 1, 2). Susceptibility indices tended to be higher in Pokhara than in the other 2 cities, but the difference was not significant (ANOVA, *F*_(2, 32)_= 1.98, *P* = 0.16). Three-point mutations in the voltage-gated sodium channel (V1016G, F1534C, and S989P) were detected in *Ae. aegypti* populations. Figures 3 and 4 show the distribution and frequencies of point mutations at 1016V and 1534F in *Ae. aegypti*. No mutation at any other loci (L982, I1011 and L1014) among the 442 *Ae. aegypti* larvae sequenced was detected. Forty homozygous and 78 heterozygous mutations of V1016G (GenBank: LC485544) were detected. The overall allelic frequency and percent homozygosity of the V1016G point mutation were 25.6% and 13.0%, respectively (Table 3). Concerning the F1534C point mutation, 6 homozygous and 24 heterozygous point mutations (GenBank: LC485546) were detected. The overall allelic frequency and percent homozygosity of the F1534 mutation were 6.0% and 2.0%, respectively (Table 3). The overall frequency of V1016G mutations was higher than that of F1534C mutations (*χ*^2^ = 86.6, *df* = 1, *P* < 0.0001). V1016G mutations were incredibly common in Kathmandu and Pokhara relative to their frequency in Bharatpur (*χ*^2^ = 83.9, *df* = 2, *P* < 0.0001). The frequency of F1534C mutations was higher in Pokhara than the other two cities (*χ*^2^ = 53.6, *df* = 2, *P* < 0.0001). A strong correlation was observed between the measured susceptibility indices and V1016G mutation frequency (*R*^2^ = 0.32, *P* = 0.0009), a pattern not observed in F1534C (*R*^2^ = 0.040, *P* = 0.28). A homozygous S989P mutation was detected in a single larva of *Ae. aegypti* collected in Pokhara (GenBank: LC485542). We detected two mutation co-occurrence patterns in 7 larvae of *Ae. aegypti*; V1016G/F1534C and V1016G/S989P. Two larvae were both a V1016G homozygote and a F1534C heterozygote, while 2 larvae were both a V1016G heterozygote and F1534C homozygotes and 3 larvae were heterozygous for both mutations. No larvae were homozygotes for both mutations (Table 4). One larva with a homozygous S989P mutation was found to be a V1016G heterozygote. Susceptibility indices in *Ae. albopictus* were lower (< 15) relative to *Ae. aegypti* in all collection sites except for one site in Pokhara (index was 18) (Fig. 5, Additional file 2: Table S2). No point mutations at L982, S989, I1011, L1014, V1016 and F1534 were detected in *Ae. albopictus* among the 359 larvae sequenced (Table 5; GenBank: LC485547 and LC485548).

**Table 1:** Susceptibility indices and allelic and homozygous percentages of mutations in the voltage-gated sodium channels in *Aedes aegypti* in Nepal

Collection Site^a^	Latitude	Longitude	Susceptibility Index	Frequency (%)								
				L982W			S989P			I1011M		
				*n*	Allelic %	Homozygous %	*n*	Allelic %	Homozygous %	*n*	Allelic %	Homozygous %
BAR001	27.63	84.50	4	na^b^	na	na	3	0	0	3	0	0
BAR002	27.62	84.54	3	8	0	0	15	0	0	15	0	0
BAR004	27.61	84.60	18	4	0	0	6	0	0	6	0	0
BAR005	27.60	84.64	3	1	0	0	4	0	0	4	0	0
BAR006	27.60	84.68	30	3	0	0	3	0	0	3	0	0
BAR007	27.58	84.73	18	8	0	0	11	0	0	12	0	0
BAR008	27.54	84.79	12	3	0	0	3	0	0	3	0	0
BAR009	27.53	84.81	5	8	0	0	13	0	0	12	0	0
BAR015	27.62	84.51	36	2	0	0	7	0	0	7	0	0
BAR018	27.73	84.45	–	2	0	0	3	0	0	3	0	0
BAR019	27.70	84.43	6	2	0	0	2	0	0	2	0	0
BAR020	27.70	84.42	15	2	0	0	6	0	0	6	0	0
BAR021	27.69	84.41	18	4	0	0	5	0	0	5	0	0
BAR022	27.69	84.42	36	7	0	0	9	0	0	9	0	0
KTM003	27.71	85.35	12	6	0	0	6	0	0	6	0	0
KTM004	27.67	85.34	12	na	na	na	1	0	0	1	0	0
KTM005	27.67	85.32	18	4	0	0	5	0	0	5	0	0
KTM006	27.75	85.35	36	2	0	0	2	0	0	2	0	0
KTM008	27.73	85.31	30	5	0	0	8	0	0	7	0	0
KTM009	27.73	85.31	18	4	0	0	6	0	0	5	0	0
KTM010	27.72	85.29	6	na	na	na	na	na	na	na	na	na
KTM011	27.71	85.28	36	1	0	0	1	0	0	1	0	0
PKR002	28.21	83.98	36	17	0	0	20	0	0	20	0	0
PKR003	28.22	83.98	36	12	0	0	16	0	0	16	0	0
PKR004	28.20	84.00	18	5	0	0	8	0	0	8	0	0
PKR005	28.20	84.01	12	3	0	0	5	0	0	5	0	0
PKR006	28.20	84.01	18	12	0	0	17	0	0	18	0	0
PKR007	28.20	84.02	18	6	0	0	8	0	0	9	0	0
PKR008	28.20	84.02	36	12	0	0	13	7.7	7.7	13	0	0
PKR009	28.21	83.99	18	9	0	0	10	0	0	12	0	0
PKR010	28.23	83.98	18	3	0	0	3	0	0	4	0	0
PKR012	28.21	83.98	30	2	0	0	2	0	0	2	0	0
PKR013	28.21	83.98	30	5	0	0	5	0	0	5	0	0

^a^Larvae were collected from 20–24 August 2017 in Kathmandu (KTM) and Bharatpur (BAR), and from 12–13 September 2018 in Pokhara (PKR)

*Abbreviation*: na, not available

**Table 2:** Susceptibility indices and allelic and homozygous percentages of mutations in the voltage-gated sodium channels in *Aedes aegypti* in Nepal

Collection site^a^	Latitude	Longitude	Susceptibility index	Frequency (%)								
				L1014F			V1016G			F1534C		
				*n*	Allelic %	Homozygous %	*n*	Allelic %	Homozygous %	*n*	Allelic %	Homozygous %
BAR001	27.63	84.50	4	3	0	0	3	0	0	3	0	0
BAR002	27.62	84.54	3	13	0	0	17	0	0	17	0	0
BAR004	27.61	84.60	18	6	0	0	7	7.1	0	19	2.6	0
BAR005	27.60	84.64	3	4	0	0	4	0	0	9	0	0
BAR006	27.60	84.68	30	3	0	0	3	33.3	33.3	4	12.5	0
BAR007	27.58	84.73	18	12	0	0	13	15.4	7.7	13	0	0
BAR008	27.54	84.79	12	3	0	0	3	0	0	5	0	0
BAR009	27.53	84.81	5	13	0	0	16	0	0	18	0	0
BAR015	27.62	84.51	36	7	0	0	11	0	0	15	0	0
BAR018	27.73	84.45	–	3	0	0	3	0	0	3	0	0
BAR019	27.70	84.43	6	2	0	0	7	0	0	7	7.1	0
BAR020	27.70	84.42	15	6	0	0	13	0	0	12	4.2	0
BAR021	27.69	84.41	18	5	0	0	15	16.7	6.7	12	0	0
BAR022	27.69	84.42	36	9	0	0	16	34.4	18.8	15	0	0
KTM003	27.71	85.35	12	6	0	0	10	50.0	40.0	12	4.2	0
KTM004	27.67	85.34	12	1	0	0	7	42.9	14.3	6	0	0
KTM005	27.67	85.32	18	5	0	0	12	29.2	0	10	10.0	0
KTM006	27.75	85.35	36	2	0	0	12	70.8	41.7	13	0	0
KTM008	27.73	85.31	30	7	0	0	13	69.2	46.2	17	0	0
KTM009	27.73	85.31	18	5	0	0	10	40.0	10.0	14	7.2	0
KTM010	27.72	85.29	6	na	na	na	na	na	na	1	0	0
KTM011	27.71	85.28	36	1	0	0	4	25.0	0	na	na	na
PKR002	28.21	83.98	36	20	0	0	19	39.5	26.3	9	66.7	66.7
PKR003	28.22	83.98	36	16	0	0	16	28.1	0	6	16.7	0
PKR004	28.20	84.00	18	8	0	0	8	50.0	25.0	5	30.0	0
PKR005	28.20	84.01	12	5	0	0	6	16.7	0	7	0	0
PKR006	28.20	84.01	18	18	0	0	18	8.3	0	17	11.8	0
PKR007	28.20	84.02	18	8	0	0	8	6.3	0	6	25.0	0
PKR008	28.20	84.02	36	13	0	0	13	26.9	15.4	5	0	0
PKR009	28.21	83.99	18	12	0	0	11	31.8	27.3	6	25.0	0
PKR010	28.23	83.98	18	4	0	0	3	50.0	33.3	3	0	0
PKR012	28.21	83.98	30	3	0	0	5	70.0	60.0	5	0	0
PKR013	28.21	83.98	30	5	0	0	5	40.0	20.0	4	0	0

^a^Larvae were collected from 20–24 August 2017 at Kathmandu (KTM) and Bharatpur (BAR), and from 12–13 September 2018 in Pokhara (PKR)

*Abbreviation*: na, not available

**Table 3:** Allelic and homozygous percentages for mutations in the voltage-gated sodium channels in *Aedes aegypti* collected in Bharatpur, Kathmandu and Pokhara

	Domain II					Domain III
	L982W	S989P	I1011M	L1014F	V1016G	F1534C
Bharatpur (*n* = 159)						
No. of samples	54	87	87	86	128	152
No. of homozygotes	0	0	0	0	6	0
No. of heterozygotes	0	0	0	0	11	4
Homozygous %	0	0	0	0	4.7	0
Allelic %	0	0	0	0	9.0	1.3
Kathmandu (*n* = 94)						
No. of samples	22	29	27	27	68	73
No. of homozygotes	0	0	0	0	17	0
No. of heterozygotes	0	0	0	0	35	5
Homozygous %	0	0	0	0	25.0	0
Allelic %	0	0	0	0	50.7	3.4
Pokhara (*n* = 189)						
No. of samples	86	107	112	112	112	73
No. of homozygotes	0	1	0	0	17	6
No. of heterozygotes	0	0	0	0	32	15
Homozygous %	0	0.9	0	0	15.2	8.2
Allelic %	0	0.9	0	0	29.5	18.5

**Table 4:** Allelic percentages of the co-occurrence of two mutations (V1016G and F1534C) in *Aedes aegypti* collected in Bharatpur, Kathmandu and Pokhara

	1534F/1534F	1534F/1534C	1534C/1534C
Bharatpur			
1016V/1016V	86.8	1.8	0
1016V/1016G	7.9	0	0
1016G/1016G	2.6	0.9	0
Kathmandu			
1016V/1016V	20.0	5.5	0
1016V/1016G	47.3	1.8	0
1016G/1016G	25.5	0	0
Pokhara			
1016V/1016V	42.9	10.2	8.2
1016V/1016G	24.5	4.1	4.1
1016G/1016G	4.1	2.0	0
Total			
1016V/1016V	60.1	4.6	1.8
1016V/1016G	21.6	1.4	0.9
1016G/1016G	8.7	0.9	0

**Table 5:** Allelic and homozygous percentages in the voltage-gated sodium channels in *Aedes albopictus* collected in Bharatpur, Kathmandu and Pokhara

	Domain II					Domain III
	L982W	S989P	I1011M	L1014F	V1016G	F1534C
Bharatpur (*n* = 181)						
No. of samples	46	67	78	77	78	83
Kathmandu (*n* = 127)						
No. of samples	59	78	76	78	78	77
Pokhara (*n* = 51)						
No. of samples	46	46	48	48	43	44

*Note*: No point mutations at L982, S989, I1011, L1014, V1016, and F1534 were detected in *Ae. albopictus* among the 359 larvae sequenced

### Linkage of point mutations with introns in *Aedes aegypti*

Two types of introns were detected between exon 20 and 21 in Nepal *Ae. aegypti* populations: group A (250 bp, GenBanK: LC485543); and group B (234 bp, GenBank: LC485541). Point mutations at 1016V (V1016G) were found to be strongly linked with the intron of group A, while such a strong linkage between point mutations at 1534F (F1534C) and the introns of group A and B was not observed (Table 6).

**Table 6:** Linkage of V1016G and F1534C mutations with the two types of intron found between exon 20 and 21 in Nepali *Aedes aegypti*

	Intron type			Statistics
	Group A^a^	Group B^a^	Group A/Group B^b^	
Point mutation at 1016V				
V1016G/V1016G	11	0	–	*χ*^2^ = 15.0, *df* = 2, *P* = 0.00056
V1016G/+	12	0	–	
+/+	14	13	–	
Point mutation at 1534F				
F1534C/F1534C	0	3	–	*χ*^2^ = 6.7, *df* = 2, *P* = 0.035
F1534C/+	2	0	–	
+/+	10	4	–	

^a^According to the classification by Martins et al. [31]; introns belonging to group A and B have length of 250 bp and 234 bp, respectively

^b^Heterozygote of the intron was not detected

### Haplotype diversity in *Aedes aegypti*

Sixty-one *Ae. aegypti* samples were successfully sequenced without the generation of double peaks, and 11 haplotypes were observed (GenBank: LC485549-LC485558 and LC489421; Additional file 1: Table S1). Four of the haplotypes had been previously reported while seven were novel. Haplotype diversity (Hd) and nucleotide diversity (π) were 0.844 and 0.004, respectively, based on calculations using the 11 haplotypes. When Hd and π were calculated using DnaSP and the 10 haplotypes of the 54 sequences (577 bp), Hd and π were 0.748 and 0.005, respectively. Tajima’s *D* was − 0.578 (*P* > 0.1), suggesting there was no evidence of population expansion. Seven samples collected in Myanmar [32] were successfully sequenced and 3 haplotypes were observed (Additional file 1: Table S1, Additional file 3: Table S3). The three haplotypes had previously been reported in earlier studies.

**Fig. 6:**
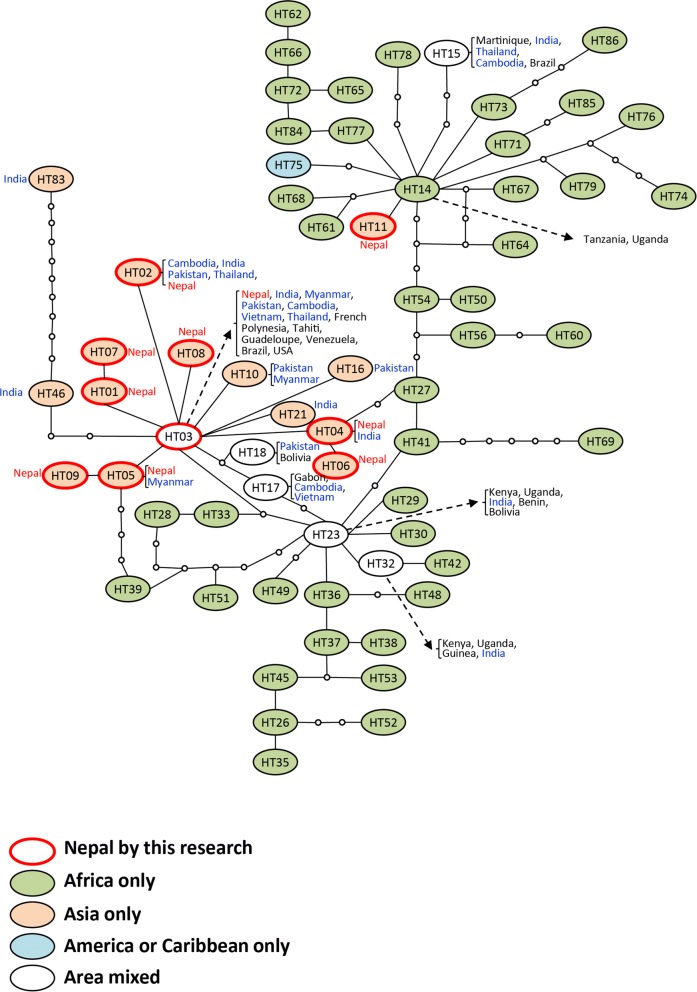
Haplotype network drawn by TCS using 64 haplotypes of length 589–593 bp. Small open circles indicate mutation steps. Oval colors indicate areas where haplotypes were reported: orange, Asia; green, Africa; blue, America; white, mixed. Ovals with red edges indicate haplotypes collected in Nepal. Countries in which haplotypes were reported are shown in the figure

Eighty-one haplotypes were identified based on 313 sequences retrieved from GenBank following sequence trimming (Additional file 1: Table S1). Among them, 5 haplotypes were identified in *Ae. aegypti* samples collected from Nepal and/or Myanmar (Additional file 3: Table S3). In total, 88 haplotypes were identified and used in TCS. The largest network consisted of 64 haplotypes of suitable lengths (589–593 bp, Fig 6). The remaining 24 haplotypes were excluded from the analysis because their sequences were either too short (404–583 bp) or their most pairwise *P*-distance was higher than 5% (HT87 and HT88). The results showed that 2 strains of *cox*1 haplotypes exist in Nepal (Fig. 6). One strain consists of 9 haplotypes derived from a globally-distributed haplotype (HT03), while the other, HT11, is derived from an African haplotype (HT14). We also trimmed sequences to 472 bp to include shorter haplotypes (e.g. the sequences detailed in Paupy et al. [33]) in the network. Because polymorphic sites were deleted by trimming, 7 haplotypes were excluded. However, 81 haplotypes were included in the largest network, similar to the previous network (Additional file 4: Figure S1).

## Discussion

In the present study, we detected a relatively high overall V1016G mutation frequency (25.6%) in *Ae. aegypti* collected from used tires in Nepal. Additionally, we detected the F1534C mutation at relatively low allelic frequency (6.0%). S989P mutations were also detected, but their overall allelic frequencies were extremely low (0.45%). Although pyrethroid resistance in *Ae. aegypti* in Nepal does not appear to be as serious an issue as it is in neighboring countries, the high frequency of V1016G mutations and its strong correlation with low pyrethroid susceptibility remains concerning. High frequencies of the V1016G mutation in *Ae. aegypti* have been reported in numerous Southeast Asian countries such as Thailand [34–36], Indonesia [37–39], Malaysia [40] and Myanmar [32]. After the first report of the F1534C point mutation in *Ae. aegypti* in Thailand [41, 42], the same mutation was subsequently found in Vietnam [43], Brazil, Venezuela, Madeira Island, Portugal [44], Grand Cayman Island [45] and Ghana [46]. F1534C mutations often co-occur with V1016G or V1016I [32, 36–38, 40, 46, 47]. Linkage of S989P and V1016G mutations is relatively common, whereas V1016G has frequently been reported in the absence of S989P. Co-occurrence of V1016G and S989P was reported to enhance the resistance to deltamethrin in *Ae. aegypti* [48]. Co-occurrence of S989P and V1016G mutations was reported to be neither additive nor synergistic; however, the S989P mutation was often found in combination with the V1016G mutation in a pyrethroid-resistant population of *Ae. aegypti* [49]. In the present study, we detected three types of co-occurrence in 2-point mutations: V1016G and F1534C, V1016G homozygous and F1534C heterozygous, V1016G heterozygous and F1534C homozygous, and heterozygous for both mutations. Interestingly, Stenhouse et al. [34] reported the different co-occurrence of the two mutations in *Ae. aegypti* populations in Thailand, in which mosquitoes of the wild type 1016V were homozygous for the F1534C mutation, 1016 heterozygous (1016V and V1016G) mosquitoes were 1534 heterozygous (1534F and F1534C), and mosquitoes with homozygous V1016G mutations were homozygous for the wild type 1534F. In Thailand, the frequency of the F1534C mutation is considerably more common than that of the V1016G mutation [34, 36, 42], a pattern which has also been reported in Vietnam [43] and Sri Lanka [50]. On the contrary, we found that the V1016G mutation was more common relative to the F1534C mutation. The relationship between the two mutations seems to be similar to that observed in Myanmar (Yangon) [32], in which V1016G mutations were dominant (84.4%) while F1534C was found at a relatively low frequency (21.2%). On the other hand, and rather interestingly, a recent report concerning *kdr* (knockdown resistance) mutations in *Ae. aegypti* in West Bengal, India, which shares its border with eastern Nepal, found a different relationship between the frequencies of the two mutations (F1534C > V1016G) [51]. Therefore, the assumption that mosquito populations depend simply on the ratio between the two mutations may be erroneous.

The linkage between *kdr* mutation (I1011M) and two types of introns positioned between exons 20 and 21, group A (250 bp) and B (234 bp), has been reported in *Ae. aegypti* in Brazil [31]. The same types of introns were also found in Ghanaian *Ae. aegypti* populations and point mutations at 1534F (F1534C) were strongly linked with the intron of group A [46]. Interestingly, in the present study, a linkage was found between V1016G and the intron of group A, while linkage between F1534C and the intron of group A was not detected. A recent study of Taiwanese *Ae. aegypti* reported a similar linkage pattern to that reported in the present study [52]. The above results may explain the genetic differences between Latin American populations (including African populations) and Asian populations of *Ae. aegypti*. The linkage between V1016G and the intron of group A was, however, not particularly strong, as the frequencies of the three haplotypes; V1016G/V1016G/intron A, V1016G/1016V/intron A, and 1016V/1016V/intron A, were almost identical (Table 6), indicating that selection pressure favoring V1016G was not strong enough.

The majority of pesticides used in Nepal are fungicides (more than 48% of total pesticides used from 2011–2012), with overall pesticide usage being very low (142 g active ingredient (a.i.) kg/ha in 2013) relative to other countries (India, 500 g a.i./ha; Japan, 12 kg a.i./ha; China, 14 kg a.i./ha, etc.). Total a.i. used in Nepal (2011–2012) was 345 tons, with pesticides used to safeguard public health (i.e. those used to control malaria and dengue fever) constituting only a small proportion [53]. The amount of insecticides used in safeguarding public health in Nepal increased from 1500 kg a.i. (2004–2005) to 3500 kg a.i. (2005–2006). This sudden increase in the use of insecticides could be attributed to the first identification of dengue fever in Nepal in 2004 [4], although since 2006, pesticide use has shown a clear and gradual decline and the total a.i. amount used in 2011–2012 was only 174 kg [53]. Sushma et al. [53] concluded that this noticeable reduction might have occurred due to the “increasing awareness of the Nepalese people regarding the harmful effects of pesticides”. The total use of insecticides in vector control reported by the World Health Organization Pesticide Evaluation Scheme (WHOPES) in Southeast Asian countries was 4251 tons a.i. in 2009 [54], among which 4000 tons a.i. was constituted by organochlorides, 164 tons a.i. was constituted by organophosphates, and 87 tons a.i. was constituted by pyrethroids. Therefore, the average amount of insecticides used by Southeast Asian countries (data from 11 countries in Southeast Asia) was 386 tons, including 14.9 tons of organophosphates and 7.9 tons of pyrethroids. These statistics emphasize the reduced use of insecticides in Nepal relative to other neighboring countries. The above data, and the fact that invasion of *Ae. aegypti* into Nepal occurred only recently, raises a simple question of the possibility of the development of pyrethroid resistance and the high frequency of *kdr* mutations in *Ae. aegypti* populations in Nepal. It might be appropriate to consider that *kdr* mutations have not been selected for in Nepal but have been introduced from neighboring countries following the invasion of mosquitoes. The rapid expansion of the primary dengue vector, *Ae. aegypti*, and dengue virus, which caused the first dengue fever outbreak in Nepal in 2006, might be attributed to the introduction of *Ae. aegypti* by humans, primarily *via* the transportation of used tires and the interchanging of people and vehicles, as well as “increased domestic breeding opportunities (increasing water storage around urban houses) due to the long-lasting water shortage” [7].

There has been a worldwide focus on the dispersal and invasion of *Aedes* mosquitoes across countries in shipping containers containing used tires that carry mosquito larvae. *Aedes albopictus* has played a leading part in this process [55, 56]. Used tire trading occurs worldwide, involving complicated commercial networks encompassing many countries. The history of the worldwide transverse expansion of *Ae. aegypti* in association with human activity may be longer than that of *Ae. albopictus*, and the vertical expansion of mosquito populations is also of significant importance in the dispersal of mosquito-borne diseases. *Aedes aegypti* larvae have been found in bamboo stumps 2130 m above sea level in the Darjeeling Himalayas [57]. Immature *Ae. aegypti* were commonly found at altitudes of up to 1700 m above sea level but are only rarely found at altitudes from 1700 to 2130 m above sea level in the highlands of Mexico. Immature *Ae. aegypti* were found in Puebla city (2133 m above sea level) [58]. Among the sites we surveyed in Nepal, the coldest collection site was Kathmandu (1200 m above sea level) located close to the Himalayan mountain range and the Siwalik hills. The temperature threshold required for the proper development of *Ae. aegypti* is reported to be 9–10 °C, with the developmental zero point being 13.3 °C [59]. Therefore, the annual minimum temperature in Kathmandu (between 11–12 °C) in 1955–1995 [60] would likely cause some difficulties during the development of *Ae. aegypti* individuals during the winter season in Kathmandu. Nayava et al. [61] reported that the average air temperature warming rate in Kathmandu was 0.063 °C/year. Assuming that the annual minimum temperature in Kathmandu in 1990 was 12 °C, the predicted minimum temperature is calculated as 12.95 °C in 2005, 13.3 °C in 2010, and 13.9 °C in 2020, according to the above rate, supporting other evidence that suggests *Ae. aegypti* was first recorded in Kathmandu in 2009 [3].

Our phylogenetic analysis using the *cox*1 gene clarified the two types of haplotype groups in Nepali *Ae. aegypti* populations; one derived from the globally-distributed haplotype group and the other from the African haplotype group. The evidence that high pyrethroid resistance and high V1016G point mutation frequencies were detected in Nepali *Ae. aegypti*, and that a smaller amount of insecticides has been used to control dengue vectors in Nepal relative to other Asian countries, which could result in a reduced selection pressure on the spread of insecticide resistance, supports the hypothesis that this species has recently been introduced from neighboring Asian countries where pyrethroid resistance is relatively widespread. The other possible pathway by which *Ae. aegypti* may have immigrated to Nepal is from African countries. Although the exact details of the relationships between Nepal and African countries are not known, the recent expansion of trading between African and Asian countries may have facilitated *Ae. aegypti* invasion.

## Conclusions

High pyrethroid resistance presumably caused by the high V1016G frequency in *Ae. aegypti* in Nepal was, to the best of our knowledge, found for the first time in the present study. The fact that *Ae. aegypti* has recently been introduced from neighboring Asian countries where pyrethroid resistance is relatively widespread was also hypothesized. As the above hypothesis were drawn by the results based on the collection data only from three cities, data sampling from the other districts might establish a more robust conclusion. The worldwide transverse and vertical invasion of vector mosquitoes in association with human activity and global warming pose significant problems for the maintenance of human public health. Moreover, the introgression of insecticide-resistance genes due to the introduction of vector mosquitoes into countries where sufficient vector control measures are not systematized might result in numerous complications in vector-borne disease control. The periodical inspection of foreign vector mosquito invasions and the evaluation of insecticide resistance in introduced vector mosquitoes are vital in the prevention of disease outbreaks and the emergence control of vector mosquitoes.

## Supplementary information

**Additional file 1: Table S1.** Assigned haplotypes, sample names, accession numbers, collection location and references for all used sequences. Samples in red letters are sequences used in the construction of the haplotype network.

**Additional file 2: Table S2.** Susceptibility indices and allelic and homozygous percentages of mutations in voltage-gated sodium channels in *Ae. albopictus* collected from used tires in Nepal.

**Additional file 3: Table S3.** Summary of haplotypes reported from each country.

**Additional file 4: Figure S1.** Haplotype network drawn by TCS using 81 haplotypes 472 bp in length. Letters in ovals are haplotype numbers. Small open circles indicate mutation steps. Oval colors indicate areas where the haplotypes were reported: orange, Asia; green, Africa; blue, America; white, mixed. Ovals with red edges indicate haplotypes collected in Nepal. Countries in which haplotypes are reported are shown.

## Data Availability

Data supporting the conclusions of this article are included within the article and its additional files. The datasets used and/or analyzed during the present study are available from the corresponding author on reasonable request. The newly generated sequences were submitted to the GenBank Database under the accession numbers LC485541-LC485558, LC489421-LC489422.

## Abbreviations

ANOVA: analysis of variance
BAR: Bharatpur
*cox*1: cytochrome *c* oxidase subunit 1
DF: dengue fever
DNA: deoxyribonucleic acid
GPS: global positioning system
Hd: haplotype diversity
*kdr*: knockdown resistance
KT_50_: time required for 50% knockdown
KTM: Kathmandu
NUMT: nuclear mitochondrial pseudogene
π: nucleotide diversity
PCR: polymerase chain reaction
PKR: Pokhara
WHOPES: World Health Organization Pesticide Evaluation Scheme
