# Supplementary figures and images for: Distribution and pyrethroid resistance status of Aedes aegypti and Aedes albopictus populations and possible phylogenetic reasons for the recent invasion of Aedes aegypti in Nepal
Source: Parasit Vectors. 2020 Apr 22;13:213. doi: 10.1186/s13071-020-04090-6 (PMC7178601; doi:10.1186/s13071-020-04090-6)

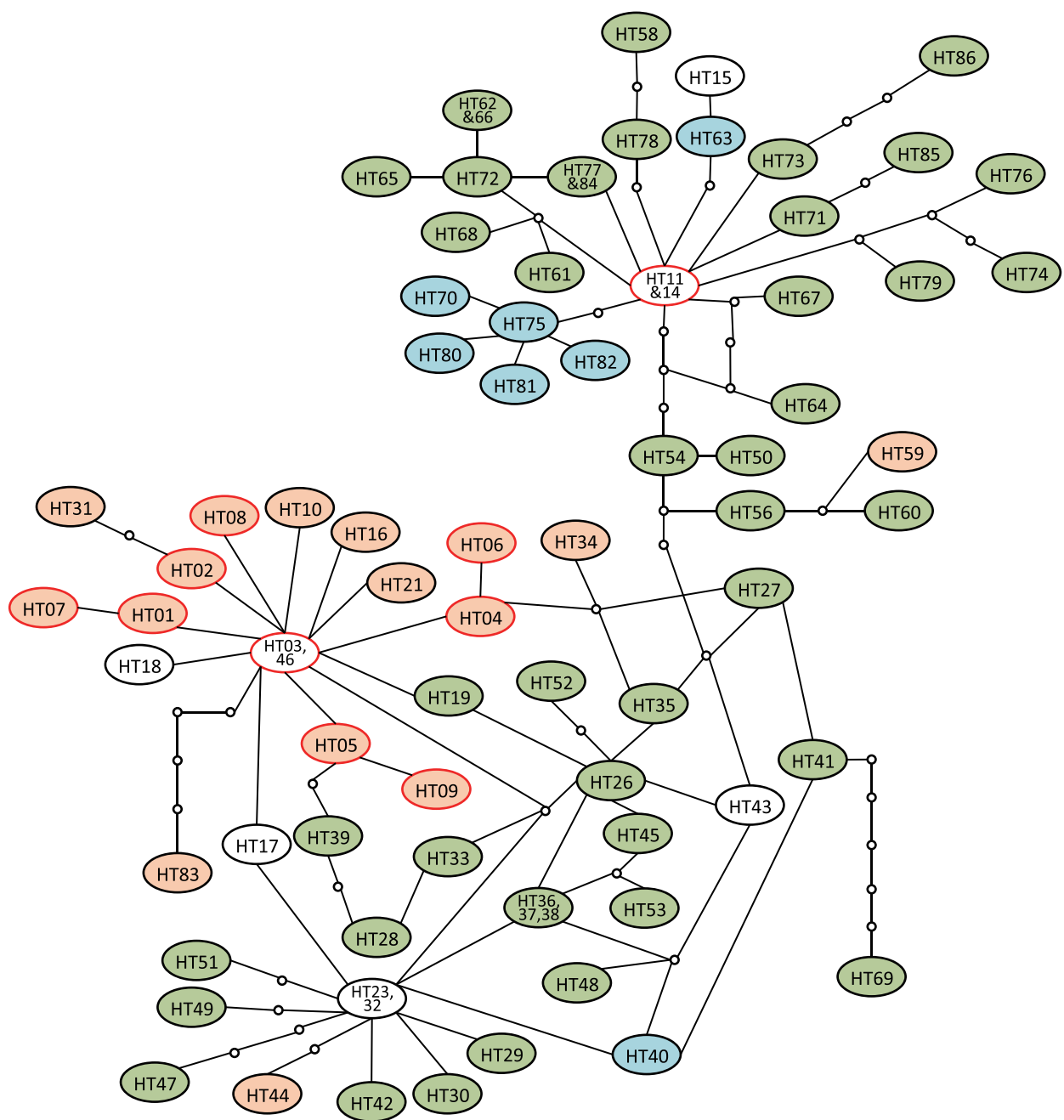

- Nepal by this research
- Africa only
- Asia only
- America or Caribbean only
- Area mixed

Supplement: Supplementary file 4 — Additional file 4: Figure S1. Haplotype network drawn by TCS using 81 haplotypes 472 bp in length. Letters in ovals are haplotype numbers. Small open circles indicate mutation steps. Oval colors indicate areas where the haplotypes were reported: orange, Asia; green, Africa; blue, America; white, mixed. Ovals with red edges indicate haplotypes collected in Nepal. Countries in which haplotypes are reported are shown. [file 13071_2020_4090_MOESM4_ESM.pdf]
